# Quality assessment in undergraduate medical training: how to bridge the gap between what we do and what we should do

**DOI:** 10.11604/pamj.2020.36.79.23658

**Published:** 2020-06-09

**Authors:** Hanneke Brits, Johan Bezuidenhout, Lynette Jean Van der Merwe

**Affiliations:** 1 Department of Family Medicine, School of Clinical Medicine, University of the Free State, Bloemfontein, South Africa,; 2 Health Sciences Education, Faculty of Health Sciences, University of the Free State, Bloemfontein, South Africa,; 3 Undergraduate Programme Management, School of Clinical Medicine, University of the Free State, Bloemfontein, South Africa

**Keywords:** Quality assessment, focus group interview, clinical competence

## Abstract

**Introduction:**

the outcome of the undergraduate medical training programme in South Africa is to produce competent medical doctors who can integrate knowledge, skills and attitudes relevant to the South African context. Training facilities have a responsibility to ensure that they perform this assessment of competence effectively and defend the results of high-stakes assessments. This study aimed to obtain qualitative data to suggest practical recommendations on best assessment practices to address the gaps between theoretical principles that inform assessment and current assessment practices.

**Methods:**

a focus group interview was used to gather this data. The teaching and learning coordinators for five of the six modules that are offered in the clinical phase of the undergraduate medical programme participated in the focus group interview. The focus group interview proceeded as planned and took 95 minutes to complete. The responses were transcribed and recorded on a matrix.

**Results:**

the lack of formal feedback to students was identified as an area of concern; feedback plays an important role to promote student learning and improve patient care. The role of teaching and learning coordinators as drivers of quality assessment were recognized and supported. All participants agreed on the outcome of the programme and the central role of the outcome in all assessments.

**Conclusion:**

the training of assessors and the implementation of workplace-based assessment and assessment portfolios were recommended and can also address feasibility challenges. Participants recommended decreasing summative assessments and only performing these for borderline students.

## Introduction

Quality assessment requires that the type and content of the assessment is aligned with the outcome of the training programme [1]. The outcome of the undergraduate medical training programme in South Africa is to produce competent medical doctors who can integrate knowledge, skills and attitudes relevant to the South African context [2]. Assessment of clinical competence is a complex process, due to a number of factors, which include the constant emergence of new best-practice medical evidence [3], the theory-practice gap between what is taught and what is observed in clinical practice [4-6], what is feasible [7], and the challenges of assessment in real-life situations that may compromise the reliability of the assessment [8]. Competence assessment must satisfy various stakeholders, which include patients and the general public, training providers, regulatory bodies and students.

Training facilities have a responsibility to ensure that they perform this assessment task effectively and can defend the results of high-stakes assessments [9]. A paper describing a framework to benchmark the quality of clinical assessment in a South African undergraduate medical programme, provides context-specific theoretical principles for undergraduate medical assessment [10]. Assessment reports and quantitative studies (In press) on current assessment practices used for undergraduate medical students at the University of the Free State (UFS) showed that these principles are not always adhered to, which may compromise the defensibility of high-stakes assessments. This study aimed to obtain qualitative data to suggest practical recommendations on best assessment practices to address the gaps between theoretical principles, that inform assessment, and current assessment practices. These recommendations will be combined with other research results to prepare a proposal to inform quality assessment at the UFS.

## Methods

**Research design:** a focus group interview (FGI) was used to triangulate theory (i.e. theoretical principles that inform assessment) with current assessment practices, to compile recommendations that should assist with quality assessment in undergraduate medical training. An FGI can be used in a mixed-methods design to triangulate qualitative and quantitative data from different sources [11], as was done in this study. Various definitions exist for an FGI, and some researchers even use the terms FGI and focus group discussion (FGD) interchangeably [12]. The difference between an FGI and an FGD is that the main objective of an FGI is to obtain answers to specific questions while, in an FGD, the interaction between the group members and the group dynamics are as important as the information gathered [12, 13].

Merton and Kendall (in Cohen *et al*.) [14] first described the concept of an FGI in 1946 and concluded that: during an FGI, there is a greater degree of interviewer control; the people participating in the interview should share experiences; the interview questions are based on previous data analysis; and subjective experiences of people who have been exposed to the same experience are gathered. The strength of a focus group is that it stimulates new or forgotten ideas and that members can build on the input of others. Some of its limitations are that it can be difficult to get members together, the group may not be representative, and some group members may dominate others [14, 15].

**Participants:** in an FGI, between five and 12 members interact, debate and argue their opinions on a specific issue. The participants of the focus group should represent the target population. Members that participate should do so voluntarily, should be knowledgeable on the subject and able to communicate in a group [11]. The clinical phase at the UFS comprises six modules. The six teaching and learning (T&L) coordinators of these modules were invited to participate in the FGI. Five of these T&L coordinators participated in the FGI.

**Facilitator:** the facilitator asks specific questions with the view to obtain answers to specific questions [13]. It is important for the facilitator to monitor the group dynamics and ensure participation by all members. The facilitator must be in control of the situation and should avoid too much or too little personal participation [12]. A facilitator with experience in higher education and in conducting FGIs was used to facilitate the process.

**Questions:** an FGI is not merely a general discussion, but is focused on a specific topic. Usually, the discussion starts broadly and, then, spirals inwards to address the research question/s [16]. The questions asked during this FGI derived from an assessment framework for undergraduate medical programmes [10], as well as the results of current assessment practices (In press) and publications with recommendations for undergraduate medical assessment [1, 2, 9, 17]. The guidelines for developing “good focus group questions”, which include that the questions must be short, clear, open-ended and directional, as described by Krueger and Casey [18] were followed. Questions were categorised and grouped. All the questions were available in the facilitator and participant guides which the facilitator and participants received before the FGI.

**Logistics:** an FGI should last between 60 and 90 minutes [19]. To capture all the information, the facilitator needs to take notes of the discussions and non-verbal cues. It can be helpful to record or videotape the discussion, and to use a co-facilitator to take notes and write down observations too [12]. The researcher arranged a neutral venue, confirmed the availability of the facilitator and participants and provided refreshments. The facilitator received all the necessary documents well in advance of the FGI. The researcher met with the facilitator in person about the process to be followed and to clarify uncertainties and agreed on the process. All participants received a participant guide one week before the FGI and a reminder to attend one day before the FGI was conducted on the 29^th^ January 2020.

**Data collection:** the aim of an FGI is not consensus, but rather the gathering of rich ideas [11]. The facilitator asked one question at a time and encouraged active participation by all participants. Discussions continued until all participants were satisfied with the answer to a particular question. If no answer or more than one answer or suggestion were offered, the facilitator encouraged participation until no new ideas were produced. More than one answer or disagreement between opinions were allowed.

**Pretesting of focus group and explorative interview:** no test run of the FGI was done, as it is important to obtain the collaborative feedback of the whole group. The validity of the questions asked in the FGI was discussed in an explorative interview with the promotors, and was based on previous experience of the researcher.

**Analysis of data and reporting:** an audio recording of the FGI was transcribed by the researcher immediately after the FGI concluded. The researcher used a video recording to verify the accuracy of the transcription. A matrix, as suggested by Onwuegbuzie *et al*. [20] was used to transfer the answers of the specific questions. Data were reported under specific categories and questions. The audio recording was used again to verify the information on the matrix.

**Ethical considerations:** ethical approval for the study was obtained from the Health Sciences Research Ethics Committee, UFS (UFS-HSD 2019/0001/2304). UFS authorities approved the inclusion of personnel. Informed consent was obtained from participants for participation and for making the audio and video recordings. Participants were not identified and a participant number was allocated to each, which is also used for data reporting.

**Quality and rigour of the data management:** to ensure the credibility of the data collection, all the research questions were clarified with the promotors. The facilitator ensured active participation by all participants, and clarified concepts to improve the quality of the data. Local, national and international assessment guidelines were included to make the recommendations transferable to other institutions. The focus group participants and interview process were clearly described for the purpose of assessing the dependability of the results. Confirmability was ensured by audio and video recording of the process and verifying results after completion of the result template.

## Results

The T&L coordinators for five of the six modules that are offered in the clinical phase of the undergraduate medical programme attended the FGI. The process proceeded as planned and took 95 minutes to complete. The audio recording was of good quality, with all conversations clearly audible and respondents identifiable. The participants provided answers to all the questions in the FGI, and all participants contributed and gave original suggestions and participated in the discussions. No participant dominated or withdrew during discussions. The results of the focus group interview are displayed in three tables according to the adjusted template suggested by Onwuegbuzie *et al*. [20]. In Table 1 the results for the outcome of the programme, competence, validity and reliability are displayed. Table 2 addresses the results for fairness, feasibility, educational effect and assessment methods and Table 3 quality assurance, training and general comments.

**Table 1:** results of the focus group interview displayed for outcome of programme, competence, validity and reliability adjusted according to the template by Onwuegbuzie *et al*.

QUESTION	ANSWERS	RESPONDENT				
		1	2	3	4	5
**1. OUTCOME OF THE PROGRAMME**						
**1.1 Do you agree with the outcome of the MBChB (Bachelors of Medicine and Bachelors of Surgery) programme?**	1.1.1 Yes	A	A	A	OS	A
	*Discussion: A clear outcome is necessary to measure the outcome of any assessment. The outcome is only visible during Internship and maybe it should be tested again then. The outcome of the programme should be kept in mind during all assessments*					
**1.2 Do you have other suggestions for the outcome?**	1.2.1 The International standard should also be included in the definition to make it more global	U	NR	A	OS	A
	*Discussion: Although it is broad, it encompasses the important concepts of competence, integration and the relevant context*					
**2. COMPETENCE**						
2.1 **Do we assess competence in the final summative assessment in the MBChB programme?**	2.1.1 Yes, but I think the bar is set too low and you pass on an average mark and may not be competent in all expected skills	U	A	A	A	OS
	*Discussion: It is difficult to achieve competence before you start to work, and Internship is also part of training and becoming competent. Students are generally competent, but I don't think that we assess competence well enough. 50% is not necessarily a mark that indicates competence. We should ensure that if a student gets 50% that the student is competent and not “half competent”, as 50% is the pass mark. This should be discussed at other forums*					
	2.1.2 I think there should be pass/fail stations or you should pass a minimum number of assessments rather than on average	A	A	OS	A	A
	*Discussion: This is a good idea. We should trust our assessment results as we do many different assessments and moderate papers*					
**3. VALIDITY**						
**3.1 To have a valid assessment enough of the content should be assessed. This can be done by blueprinting of all assessments. How can we improve blueprinting of all assessments?**	3.1.1 We should start sooner by planning and blueprinting all tests and assessments and not only exams.	NR	A	A	A	OS
	*Discussion: I want to emphasize that blueprinting is making life much easier. I luckily inherited the system, but then had to get some training as well. Information leaflets on Blackboard may help especially with turnover of personnel*					
	3.1.2. All T & L coordinators should do the Health Professions Education assessment course	A	A	A	OS	NR
	3.1.3 T & L coordinators should lead the process in departments to implement blueprinting of all assessments	A	A	OS	A	A
	3.1.4 Informal blueprinting happens, but it should be formalized to have evidence	A	OS	A	A	A
**3.2 Students and lecturers think that assessment methods should be improved. What methods can you suggest to improve assessment?**	3.2.1. We use all methods and I don't think we need to add methods, however it is difficult with limited workforce	A	A	OS	A	A
	*Discussion: It is easier to use MCQ's than longer questions, although higher cognitive levels may be difficult to assess. The workforce is the problem*					
	3.2.2. Continuous and portfolio assessment may be a way to go and we should work towards it	U	A	A	OS	A
	*Discussion: Continuous assessment is problematic, due to assessors not giving marks or giving 65%. The number of assessments may be beneficial*					
	3.2.3 Longer questions may test concepts better	OS	A	U	NR	A
	3.2.4.The methods are good, the assessors not always and they may benefit from rubrics and training	A	A	OS	A	A
	*Discussion: Assessors need to be trained better, e.g. registrars can be trained in assessment, by allowing them to assess together with a consultant and then discuss the marks. It will benefit both parties and address the work force*.					
**3.3 According to the Health Professions Council of South Africa (HPCSA) “soft skills and professionalism” should be assessed. How do you suggest that we assess "soft skills" and professionalism throughout the curriculum?**	3.3.1 Soft skills are assessed in clinical case presentations, but a specific mark is not allocated to it. We may allocate a specific mark to it	A	A	OS	NR	A
	3.3.2 In communications stations it can be assessed as well	NR	A	A	OS	NR
	*Discussion: Assessments are not normal circumstances and students know how to behave professionally in assessments, however professionalism should be practiced and assessed throughout. I like the idea of peer and patient assessment. Peer review may not work, it was tried before. Unprofessional behaviour should be recorded and have consequences e.g. deduction of marks. A “Professional portfolio” for continuous assessment may work. Facilitator: The university is involved in a programme to promote graduate attributes and we may look how they do it and what is in place from their side*					
	3.3.3 We should try and latch to the university programme	A	OS	A	A	A
**4. RELIABILITY**						
**4.1 Can/Should all assessments be 100% reliable?**	4.1.1 No, it is impossible	A	A	A	A	OS
	4.1.2. But we should try to keep it as reliable as possible, taking the real-life situation into account	A	A	A	OS	A
**4.2 Which specific measures can be implemented to improve or get reasonable reliability in clinical assessment?**	4.2.1 We should use more clinical cases in the workplace (WBA), which is less labour intensive than an exam	U	A	U	OS	A
	*Discussion: It is difficult with the limited resources. Assessment rubrics may make it easier to implement, as well as simulated scenarios*					

Codes: A -Agree, D -Disagree, U -Uncertain, NR -No response, OS -Original suggestion

**Table 2:** results of the focus group interview displayed for fairness, feasibility, educational effect and assessment methods adjusted according to the template by Onwuegbuzie *et al*.

5. FAIRNESS						
**5.1 How can we improve the alignment between outcomes, training and assessment?**	5.1.1 Lecturers should be asked to update “outcomes” yearly in line with clinical practice and assessment experience, before the new groups start	NR	A	OS	A	A
	5.1.2 Student feedback of the module should also be considered	A	A	NR	A	OS
	*Discussion: The outcomes should be a framework, rather than specific. This is tricky, because we need specifics to blueprint. The students struggle with the transition between pre-clinical with specific outcomes and clinical training with broader outcomes. They need to mature in this regard. These are senior students and we should not spoon feed them. They must be able to integrate and think rather than concentrating on detail*					
	5.1.3 T & L coordinators must facilitate the process to ensure alignment and fairness	A	A	A	A	OS
**6. FEASIBILITY**						
**6.1 Which resources do you take into account when planning individual and overall assessments?**	6.1.1 The basics are assessors, timing and patients. The numbers are calculated according to the number of students	A	A	OS	A	A
	*Discussion: This is more difficult with the addition of training sites, increased student numbers and the Nelson Mandela Fidel Castro Medical Programme (NMFCMP) students*					
**6.2 How important is each one of those?**	6.2.1 The assessors, timing and patients are most important	A	A	A	OS	A
	6.2.2 Recently finances must also be considered.	NR	A	OS	NR	A
	*Discussion: Patients must come in for exams and also wants compensation, transport money and food. Travel and/or accommodation of External examiners must also be budgeted for. This adds up to a substantial amount. Less summative assessment may help with resources*					
**7. EDUCATIONAL EFFECT**						
**7.1 Feedback is one of the most important aspects of learning. What strategies can be used to ensure effective feedback?**	7.1.1 Logistically it is difficult because students start in a new rotation. It may help if a specific session is scheduled on the time tables, say 2 weeks into the new rotation	A	A	A	A	OS
	7.1.2 Electronic feedback to the group via e-mail or on Blackboard	OS	A	A	OS	A
	*Discussion: Information can include the class average and highlighting of problem areas*					
	7.1.3 Appointments with individual students who struggled with the assessment	A	OS	A	A	A
	7.1.4 Open door policy to come and discuss the assessment with the T & L coordinator, as is currently the practice	A	A	A	A	OS
	7.1.5 Immediate feedback after clinical cases to highlight strengths and areas that need improvement.	OS	A	A	A	A
**7.2 How should feedback be given on multiple choice questions?**	7.2.1 This is difficult, because we don´t want to compromise our databank. However general feedback is given on problem areas after the assessment.	OS	A	A	A	A
	*Discussion: Students want the answers, rather than the knowledge and therefor general feedback is given*.					
	7.2.2 The students may re-write the test under exam conditions and then the answers are discussed	A	A	A	OS	NR
	7.2.3 Poor performers may come and have a look at their paper in order to identify the root of the problem.	U	OS	U	A	A
**8. TYPES OF ASSESSMENT**						
**8.1 Students and some lecturers suggested only end-of-block assessments and if students pass they need not do a summative assessment at the end of the year again. How do you feel about this suggestion?**	8.1.1 It is a good idea and practiced at other universities.	NR	A	A	A	OS
	*Discussion: It motivates students to work hard during rotations. A pass mark of 60% was agreed upon. This will ensure that borderline candidates can be assessed again during summative assessment*.					
**8.2 What is your opinion on a single integrated assessment?**	8.2.1 I fully support it, like the Family Medicine OSCE and then all can contribute to the assessment	NR	OS	A	A	A
**8.3 With guidelines for good assessment practices in mind, how can we improve our current assessment regarding Workplace-based assessment (WBA)?**	8.3.1 This is the ideal way forward and we must try to implement it, despite workforce problems.	U	A	A	OS	A
	*Discussion: This is the best place to assess real-life competence. The students, patients and assessors are there. Peer assessment may be problematic. The use of a “competency portfolio” was suggested and supported*.					
**8.4 How can we improve formative assessment?**	8.4.1We should try to assess and record more student patient encounters	A	A	A	A	OS
	*Discussion: This will increase the number and the reliability of assessments. You know which students are competent when you work with them. You can also assess professionalism better. The competency portfolio was mentioned again*.					
**8.5. What is your opinion on summative assessment?**	Although more assessments are good learning opportunities, I think we must try and reduce summative assessment to only borderline candidates.	A	OS	A	A	A
	*Discussion: Students may be disadvantaged during their first rotation, because they gain experience and competence throughout the year. However all will have first rotations and it is therefore fair*.					

Codes: A -Agree, D -Disagree, U -Uncertain, NR -No response, OS -Original suggestion

**Table 3:** results of the focus group interview displayed for quality assurance, training and general comments adjusted according to the template by Onwuegbuzie *et al*.

9. QUALITY ASSURANCE						
**9.1 What strategies should be implemented to ensure compliance with the UFS pre- and post-assessment moderation practices?**	9.1.1 Although it is more, work a moderation checklist should be implemented for all assessments	A	A	OS	A	A
	*Discussion: It is done informally, without any evidence when needed, therefore it should be formalized. Quality assurance helps to improve assessments and maintain standards*.					
**10. TRAINING**						
**10.1 Students suggested more exposure to patients in wards and clinics and less in the classroom. What are your suggestions to improve clinical training?**	10.1 1. With more students (and less lecturers) the direct student exposure decreases. Time at training sites should be stipulated and controlled.	OS	A	A	A	A
	*Discussion: Most students want to go home as soon as possible, stating that they want to study, which is contradicting what they suggested*.					
**10.2 Students also suggested more exposure to good clinical role models. What is your response to this?**	10.2.1 All clinicians are not necessarily good role models, but students can also learn from the “not so good” on what not to do.	OS	A	A	A	A
	*Discussion: Due to workload many people are suffering from burnout. This must also be discussed with students and the importance of self-care must be re-emphasized. The clinical psychologist can assist and attention should be paid to resilience training. Reflective practice and professionalism must also be addressed. Students get very good support at the UFS to cope with stress*.					
**10.3 Students want to be assessed on what they see during training, but not at specialist hospitals. What is your response to this?**	10.3.1 Students get exposure to all levels of care to expose them to different conditions and clinical signs. Students must know what is expected where, to benefit from the extended training platform.	A	A	A	OS	A
	*Discussion: In tertiary hospitals the clinical signs are more obvious. At primary care, they get exposure to the burden of disease in their environment. Students are not expected to make specialist diagnosis, but rather display their approach to a specific symptom or sign. Supervision and space is problematic at primary care clinics. Good communication is important to know what is expected*.					
**11. GENERAL**						
**11.1 Any last comments regarding the quality of undergraduate clinical assessment and training?**	11.1.1 We, the T & L coordinators do a great job under difficult circumstances	A	A	A	OS	A
	11.1.2 The T & L coordinators made a huge difference to the quality of assessment and training	OS	A	A	A	A

Codes: A-Agree, D-Disagree, U-Uncertain, NR-No response, OS-Original suggestion

## Discussion

The FGI met the requirements for a good FGI regarding participants, the facilitator, the questions, logistics, explorative interview and data collection and analysis. The results are also representative of the study population, with five of the possible six participants included. The first question was around the outcome of die undergraduate medical programme. All the participants agreed with the outcome as is, namely, to produce a competent medical doctor who can integrate knowledge, skills and attitudes relevant to the South African context. This clear outcome should be kept in mind during all assessments. This outcome is in line with the regulations stipulated in the Health Professions Act of South Africa, the South African Quality Assurance Authority and the assessment policy of the UFS [2, 17, 21].

The next questions focused on competence and the way it is assessed. Clinical competence must be assessed on the “Does” level, according to Miller's pyramid [22]. It was mentioned that the actual demonstration of this competence only occurs during internship, which is still part of training (students must complete internship and community service before registering as independent medical practitioners with the Health Professions Council of South Africa (HPCSA). A suggestion to implement pass/fail stations and not only an average of 50% or above to pass, was well accepted. A discussion on the difficulty to ensure competence with a pass mark of 50% (the pass mark according to the UFS assessment policy) provided more questions than answers. It must be recognised that a mark of 50% indicate that the student is competent and not “half competent”. All assessors should be aware of how they allocate marks and the implication thereof. Further discussion in this regard was recommended to clarify the meaning of 50% in the context of competence.

During questions regarding validity, good practices were shared and recommendations made. It was agreed that T&L coordinators should take responsibility for assessments, to ensure the validity of assessments. Blueprinting of all assessments should be done. Blueprinting will improve content validity, and using appropriate assessment methods will improve construct validity [10]. There is no need to add additional assessment methods, as most assessment methods described for undergraduate clinical assessment [23] are currently used at the UFS. It was recognized that a shortage in the workforce favours the use of less labour-intensive assessment methods, e.g. multiple-choice questions rather than longer written questions that can assess higher cognitive levels. The lack of trained assessors also limits the use of workplace-based assessment (WBA) and assessment/competency portfolios to assess competence. To address the workforce issue, all clinicians should be trained as assessors, and registrars can be included in the assessment process. By including registrars, they are trained on the important skill of assessment, and it may help to spread the workload. Regarding the assessment of professionalism and “soft skills,” the suggestion to implement a “professionalism portfolio” and implement the graduate attributes policy of the university were supported and should be investigated.

The participants gave valuable input on aspects to improve the quality of assessment, including recommendations on reliability, fairness, educational effect and feasibility. Competency assessment cannot be 100% reliable, but the suggestions to use WBA and assessment/competency portfolios were recommended to increase the number of assessments. WBA and assessment portfolios are excellent ways to assess competence, but reliability may be compromised [24]. Although portfolios and WBA are labour intensive, these methods are more authentic and the number and type of assessments can increase, thereby contributing to reliability [25]. The lack of formal feedback to students was identified as an area of concern - feedback plays an important role to promote student learning and improve patient care [26]. Feedback is also a requirement stipulated in the assessment policy of the UFS [21]. The scheduling of formal feedback sessions after assessments may assist with the implementation of formal feedback, a practice that is currently lacking in the undergraduate medical programme.

Participants in the FGI recommended decreasing summative assessments and only performing these for borderline students. This practice will also address some of the problems with the feasibility of summative assessments. Less emphasis on summative assessment is well supported in the literature e.g. assessment results should not depend on a single summative assessment, as competency in one case is a poor predictor of competency in another [27]. Performance stress during high-stakes assessments may also contribute to less reliable outcomes [28], and a single poor performance should not affect the outcome of years of training [23]. The lack of post-assessment moderation was identified as a risk for quality assessment. Although procedures and checklists for moderation are available, the implementation is not standard practice in all departments. Quality assurance and moderation are important components of ensuring and maintaining the quality of assessment [21]. An e-mail to remind departments to do moderation, and spot checks, may reinforce the implementation of this important practice.

During the FGI, clinical training was also discussed in relation to assessment. Biggs [29] describes the term constructive alignment as comprising outcomes, teaching and training activities and assessment that are planned to complement and support each other. Students indicated in their feedback before the FGI that they want more on-site practical training in wards and clinics (In press). The increase in student numbers and decrease in teacher numbers also decreases supervised, hands-on practical training for students. A suggestion for countering the lack of clinical exposure is to stipulate clearly and monitor available clinical training time. Another factor that affects clinical training negatively is overburdened clinicians, who may not necessarily be good role models and tend to give students time off, so that the clinicians can get clinical work done, rather than spend time on training. This practice may be due to burnout, as evidenced by a study in this academic setting that showed that only 3.4% of the doctors included in the study showed no signs of burnout [30]. The participants mentioned the importance of developing core competencies in undergraduate students, such as professionalism, leadership and scholarship [31], how to cope in difficult situations, and practicing self-care which should be included in clinical training.

The training platform may be an opportunity for students to see how to behave professionally, but also how not to behave. It was discussed that students may not be aware that, although they are trained in tertiary facilities, they are not expected to perform as specialists, but that they should rather use the opportunity to identify clinical signs and develop an approach to a specific problem. Better communication on the outcome of specific training rotations may assist both students and clinicians and was recommended. The FGI concluded with a discussion on the effect of the introduction of T&L coordinators on student assessment and training. The excellent work of the T&L coordinators was recognised and appreciated. All agreed that the T&L coordinators should continue to play a leading role in student assessment and training.

**Limitations and strengths:** only the T&L coordinators of the major disciplines participated in the FGI, and the FGI may have failed to capture contributions by excluding minor disciplines. However, these smaller disciplines were indirectly represented by the major disciplines. Strengths of the FGI were that the FGI was conducted according to the planning, and within the guidelines for a FGI, as described in the methods, and that data management met the criteria for credibility.

## Conclusion

The clear, agreed-upon outcome, namely, to produce a competent medical doctor who can integrate knowledge, skills and attitudes relevant to the South African context, should be kept in mind during all assessments. The difficulty of how to measure and allocate marks to competence was recognised. The lack of formal feedback to students and blueprinting should be addressed. The important place of WBA and assessment portfolios, with less emphasis on summative assessment were important recommendations from the FGI. A proposal to improve the quality of assessment in the clinical phase of the undergraduate medical programme will be compiled from this and other research information. This proposal will be submitted to the Executive Committee of the School of Clinical Medicine for implementation. Finally, an FGI can be recommended as an appropriate way to get rich data for practical solutions.

### What is known about this topic

- Assessment should be aligned with the outcome of the training programme;
- Assessment of clinical competence is a complex process.

### What this study adds

- Workplace-based assessment should form part of competency assessment;
- The difficulty of how to measure and allocate marks to competence was recognised;
- Competency and professional portfolios should be implemented.
